# Cx40 Suppresses Sprouting Angiogenesis *In Vitro*

**DOI:** 10.1089/bioe.2023.0034

**Published:** 2023-12-15

**Authors:** Edward K. Looker, Femke J. Aan, Christopher J. Hatch, Christopher C.W. Hughes, Michelle L. Matter, Jennifer S. Fang

**Affiliations:** ^1^ Department of Cell and Molecular Biology, School of Science and Engineering, Tulane University, New Orleans, Louisiana, USA.; ^2^ Department of Biochemistry and Molecular Biology, School of Medicine, Tulane University, New Orleans, Louisiana, USA.; ^3^ Department of Biomedical Engineering, Henry Samueli School of Engineering, University of California-Irvine, Irvine, California, USA.; ^4^ Department of Molecular Biology and Biochemistry, School of Biological Sciences, University of California-Irvine, Irvine, California, USA.

**Keywords:** Cx40, Cx43, Cx37, connexins, gap junctions, blood vessels, endothelial cells, sprouting angiogenesis, cell proliferation, cell migration, organ-on-a-chip, microphysiological models, bead assay

## Abstract

Blood vessels are highly organized and form during development through a series of complex processes that include vasculogenesis, sprouting angiogenesis, and vessel remodeling. Several gap junction proteins (termed connexins, Cx)—including Cx40 (GJA5)—are expressed in vascular endothelium early during vessel development and are critical for establishment of a healthy vasculature. However, Cx40's specific role in regulating vessel growth remains uncertain: while previous studies have shown that developmental and cancer-associated neovascularization is reduced in Cx40-knockout mice, Cx40 knockout in zebrafish embryos enhances intersegmental vessel growth. Thus, in the current study, our aim was to identify Cx40's specific role in sprouting angiogenesis. First, we used a vessel-on-a-chip microphysiological model to confirm Cx40's overall necessity for microvessel network development. Next, we used the fibrin gel bead assay—a three-dimensional *in vitro* model of sprouting angiogenesis—to assess Cx40's necessity for this process. We found that Cx40 knockdown in endothelial cells (EC) drives more aggressive sprouting angiogenesis in association with increased EC proliferation. By contrast, using electrical cell-substrate impedance sensing we observed no effect of Cx40 knockdown on EC migration. Finally, we found that Cx37 (GJA4) is reduced in Cx40-deficient EC and that targeted silencing of Cx37 alone produces a more aggressive, hypersprouting phenotype compared to control or Cx40 knockdown EC. Taken together, our data indicate that Cx40 plays multiple roles during vessel growth, including to specifically limit sprouting angiogenesis, and that this may occur, at least in part, through regulation of endothelial Cx37 levels.

## Introduction

Blood vessels form during development through vasculogenesis, sprouting angiogenesis, and vessel remodeling. Vasculogenesis describes the coalescence of individual endothelial cells (EC) into a *de novo* primitive tubular network. Sprouting angiogenesis involves expansion of the existing vascular plexus through formation of invasive angiogenic sprouts composed of specialized tip and stalk cells. Vascular remodeling describes several complex processes, including EC quiescence, arteriovenous specification, mural cell recruitment and investment, vessel bed reorganization, and vessel regression and pruning. Most of these processes are recapitulated in the postnatal animal to support new vessel growth during wound healing and tissue revascularization, and all likely rely on tightly controlled cell–cell communication to coordinate these processes, including through intercellular gap junction channels that mediate electrochemical coupling between adjacent cells.

Vascular endothelium expresses several gap junction proteins—termed connexins (Cx)—including Cx37, Cx40, and Cx43 beginning early in vascular development.^1^ Of these, Cx37 and Cx40 are initially abundant but become increasingly restricted to the larger vessels of the established vasculature.^2–4^ There, both Cx37 and Cx40 expression are shear sensitive^5,6^ and required for arteriovenous specification, arteriogenesis, and vessel function.^5,7^ Single knockout of Cx37 or Cx40 produces viable mice with distinct vascular phenotypes^8,9^ and only when both Cx are knocked out in combination do animals fail to survive past birth,^10^ suggesting that both play distinct, as well as likely partially redundant or overlapping, roles in the vasculature.

Specifically, Cx40 supports endothelial cell-cell communication of signals that control vessel tone,^11–13^ whereas Cx37 is dispensable for this process^11^ and instead controls EC quiescence and the remodeling response.^7,14^ More recently, Cx40 has been shown to also regulate vessel growth and remodeling; yet, it remains uncertain exactly how Cx40 is involved in this process: for example, while microvessel density is reduced in Cx40^−/−^ mice during development and in cancer models,^15,16^ knockout of both Cx40 orthologs (Cx41.8 and Cx45.6) in zebrafish produces a striking *hypersprouting* phenotype characterized by increased growth rate of intersegmental vessels.^6^ Thus, the question of whether Cx40 drives or limits vascularization—or possibly, both—remains unclear from these previous studies of intact animals.

*In vitro* models may be able to provide some insight into this question. Previous groups have shown that knockdown of Cx40 is necessary and sufficient for endothelial cord formation in an *in vitro* endothelial tube-forming assay,^17,18^ which primarily models vasculogenesis. In the current work, our aim was to complement these earlier studies using fully-humanized *in vitro* models to measure Cx40's specific effects on vessel organization and sprouting angiogenesis. First, we assessed requirement of Cx40 for overall microvessel network formation in the vascularized micro-organ (VMO) device, a recently-established vessel-on-a-chip microfluidic platform that captures multiple processes of vessel growth and remodeling in a microphysiological, three-dimensional *in vitro* setting under flow.^19,20^

Next, we used the fibrin gel bead assay (“Bead Assay”)—an *in vitro* model that specifically captures tip/stalk cell dynamics in sprouting angiogenesis^21–23^—to test Cx40's specific requirement for sprouting angiogenesis, where we found that (consistent with observations in zebrafish^6^ and counterintuitive with regard to the overall effect of Cx40-deficiency on microvessel complexity) Cx40 suppresses sprouting angiogenesis.

## Materials and Methods

### Cell culture

Primary human umbilical vein EC were purchased (Lonza) or isolated in-house as previously described.^24^ EC were cultured on gelatinized surfaces in complete EGM2 (Lonza). In some experiments, EC were transduced with lentivirus to induce constitutive expression of a fluorescent reporter protein (e.g., Venus, Azurite, or mCherry) at an multiplicity of infection ≈2. Primary human lung fibroblasts (Lonza) were cultured in 10% Dulbecco's modified Eagle's medium (Corning) supplemented with 50 μg/mL gentamicin. All primary cell types were discarded after passage 8. For gene knockdown studies, cells were plated at ∼60% confluence and transfected with 10–30 nM scrambled control (si-Ctrl) (Dharmacon or Ambion), or with si-Cx40 or si-Cx37 (Dharmacon SMARTpool) using Lipofectamine 2000 (Sigma) or Lipofectamine RNAiMax (Sigma) according to manufacturers' protocols. Cells were then allowed to recover for 24–48 h before functional assays.

### Vascularized micro-organ

VMO devices were loaded as previously described.^19,20^ In brief, fluorescent EC were co-seeded at a 1:1 ratio with primary human fibroblasts into an 8 mg/mL fibrin hydrogel within a VMO microfluidic device, and gravity driven interstitial flow of complete EGM2 was established. Network formation was monitored over 10 days by fluorescent imaging (Zeiss). Vessel morphometry was performed using the AngioTool plugin^25^ for Fiji/ImageJ.

### Fibrin gel bead assay

Fibrin gel bead assays (“Bead Assay”) were performed as previously described.^21–23^ In brief, EC were cultured on the surface of collagen-coated beads (Cytodex 3, Amersham; or SphereCol, Sigma) and embedded into 2.5 mg/mL fibrin hydrogels. Primary human fibroblasts were then cultured on top of the hydrogel at a density of 20,000 fibroblasts/well of a 24-well plate. Sprout formation was monitored by phase imaging (Nikon), epifluorescence (Nikon, Zeiss), or confocal imaging (Leica SP10). Sprout morphometry was performed manually using Fiji/ImageJ, including quantification of sprout number (i.e., mean sprout number per bead), branching index (i.e., mean number of branches per sprout for each bead), sprout length (i.e., mean sprout distance from bead surface), and sprout diameter (for each sprout, when measured at the bead surface).

### Quantitative polymerase chain reaction

RNA was isolated (Quick-RNA Microprep Kit, Zymo) and used to synthesize cDNA libraries (iScript cDNA Synthesis kit, BioRad). SYBR Green-based quantitative polymerase chain reaction (qPCR) was performed using a Bio-Rad CFX system with the real-time qPCR primers listed in Table 1, and with an upper threshold cycle number (C_q_) value set to 45. In experiments in which genes could be detected below threshold in control but not knockdown groups, fold expression following knockdown was set to 0.

**Table 1.** Quantitative Polymerase Chain Reaction Primers

Gene name	Forward primer	Reverse primer
18S	CCCCGGCCGTCCCTCTTA	CGCCCCCTCGATGCTCTTAG
Cx37 (GJA4)	ACACCCACCCTGGTCTACC	CACTGGCGACATAGGTGCC
Cx40 (GJA5)	CCGTGGTAGGCAAGGTCTG	ATCACACCGGAAATCAGCCTG
Cx43 (GJA1)	GGTGACTGGAGCGCCTTAG	GCGCACATGAGAGATTGGGA

Real-time qPCR primers used in this study are listed below in 5′-3′ orientation.

qPCR, quantitative polymerase chain reaction.

### Immunofluorescence

Silencing RNA (siRNA)-transfected EC monolayers were cultured on gelatinized glass coverslips in complete EGM2 media before fixation with 4% paraformaldehyde. Following 1 h of block (3% bovine serum albumin [BSA] 0.1% Triton X in phosphate-buffered saline [PBS]), cells were incubated in primary antibody overnight at 4°C (1:100 in 1% BSA 0.1% Triton X in PBS). After wash, cells were then incubated for 2 h at room temperature in fluorescently-conjugated secondary antibody (Alexa594-conjugated donkey anti-rabbit [H+L], 1:600; Fisher). Nuclei were stained with 15-min incubation in 10 μg/mL Hoechst 33342 (Sigma) and mounted on glass slides for imaging on a Leica DMi8 microscope.

### Cell proliferation assay

Subconfluent siRNA-transfected EC monolayers were cultured in complete EGM2 media and exposed to a 2.5 h pulse of 20 μM 5-ethyl-2′-deoxyuridine (EdU) before cell fixation and staining using a Click-iT EdU Cell Proliferation Kit (Thermo) according to manufacturer's protocol.

### Electrical cell-substrate impedance sensing

Electrical cell-substrate impedance sensing (ECIS) was used to measure EC migration following monolayer wounding using a 1600R ECIS instrument (Applied Biophysics), as previously described.^26,27^ In brief, siRNA-transfected EC monolayers were cultured as confluent monolayers into each well of a gelatinized 8-well PET chip containing a single 0.05 mm^2^ electrode per well (8W1E; Applied Biophysics). Following baseline recording of monolayer impedance, cell wounding was performed with the 1600R ECIS instrument (Applied Biophysics) by means of 20-sec administration of 1400 μA wound current at 60 kHz at *t* = 0.5 h. Dead cells were removed with a rinse of complete EGM2 media. Monolayer impedance was then measured continuously to monitor monolayer impedance recovery over time due to cell migration into the monolayer wound back to baseline. Migration rate was determined using the following calculations: migration rate = electrode radius (μm)/Δt (h); Δt = t_2–_t_1_ where t_1_ is the timepoint (h) at which cells were first wounded and t_2_ is the time point (h) at which point monolayer resistance returns to baseline.

### RNA sequencing

Transcriptomic sequencing of siRNA-transfected EC was performed using an Illumina NovaSeq 6000 through the University of California-Irvine Genomics, Research and Technology Hub. FASTQ files underwent trimming of adapter regions and removal of low-quality reads with fastp.^28^ GRCh38 reference genome was built using bowtie2^29^ from Ensembl release 105,^30^ and FASTQ files were aligned to reference genome using STAR.^31^ FASTQ files were merged using SAMtools^32^ and a count matrix of exon protein coding genes was generated using featureCounts.^33^ The count matrix was imported into RStudio (RStudio Team (2020). RStudio: Integrated Development for R. RStudio, PBC, Boston, MA; www.rstudio.com/) running R (version 4.0.3 [R Core Team (2020). R: A language and environment for statistical computing. R Foundation for Statistical Computing, Vienna, Austria; https://www.R-project.org/]), and differentially-expressed genes (DEGs) for each condition were determined using DESeq2.^34^ Gene ontology (GO) term analysis was performed using the online PANTHER knowledgebase.^35^

### Statistics

Statistical comparisons were performed using either Student's *t*-test (parametric data) or Wilcoxon Rank-Sum test (nonparametric data), or one-way analysis of variance (ANOVA) for multiple comparisons followed by *post hoc* Student's *t*-test. For the competition Bead Assay, tip cell distribution was compared using a chi-square test. Statistically significant comparisons are denoted whenever *p*-values were calculated to be <0.05.

### Resource sharing

Differential gene expression list generated through RNA sequencing has been made available as Supplementary Data S1 and will be deposited in the National Center for Biotechnology Information (NCBI) Gene Expression Omnibus (GEO) database (GSE243404). VMO data will be deposited through BioSystics-AP, a database for microphysiological systems data sharing.^36^ All other data will be made available through Figshare.

## Results

### Cx40 knockdown reduces microvessel complexity in a vessel-on-a-chip platform

**FIG. 1.**
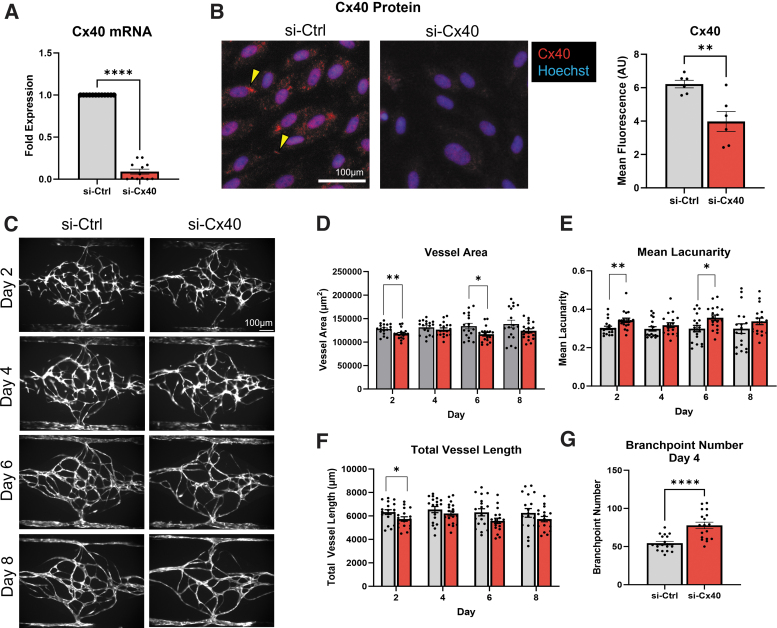
Cx40 promotes microvessel network formation in an organ-on-a-chip platform. **(A)** siRNA (si-Cx40) knocks down endogenous Cx40 mRNA transcript expression by >90% in EC. **(B)** Total and junctional (yellow arrowheads) Cx40 protein levels are reduced in si-Cx40 HUVEC monolayers versus si-Ctrl, and are quantified as a significant decrease in total Cx40 mean fluorescence (AU). **(C)** si-Ctrl and si-Cx40 EC form networks in the VMO platform, where si-Cx40 networks display **(D)** reduced vessel area and **(E)** increased mean lacunarity at days 2 and 6. **(F)** Total vessel length was also reduced at day 2 in si-Cx40 networks. **(G)** Branch point number was transiently increased in si-Cx40 networks at day 4. *p < 0.05, **p < 0.01, ****p < 0.0001. AU, arbitrary units; EC, endothelial cells; HUVEC, human umbilical vein endothelial cells; si-Ctrl, scrambled control; siRNA, silencing RNA; VMO, vascularized micro-organ.

Previous investigators have reported that vessel density is reduced in the neonatal mouse retina, adult heart, and in tumor xenograft models.^15,16,18^ To confirm effects of Cx40-deficiency on vessel development in an *in vitro* setting, we first used siRNA to selectively target endogenous Cx40 transcript in human umbilical vein EC. We achieved on average >90% knockdown of endogenous Cx40 mRNA compared to EC transfected with si-Ctrl (Fig. 1A). We then confirmed that si-Cx40 transfection also reduced endogenous Cx40 protein levels in cultured EC monolayers (Fig. 1B).

Next, we seeded si-Ctrl or si-Cx40 EC (engineered to constitutively express a fluorescent reporter protein) into the VMO device, a vessel-on-a-chip platform in which lumenized and perfusable microvessels develop over the course of 7 days in a three-dimensional hydrogel under flow.^19,20^ This microphysiological model recapitulates *in vivo* vessel growth—including vasculogenesis, angiogenesis, and vessel remodeling—and includes several hallmarks of intact tissue, including perivascular support cells, extracellular matrix, and circulation of a blood substitute through lumenized microvessels.^19,20^ Importantly, this model allows us to dissect away systemic cardiovascular effects of Cx40 ablation—for example, chronic hypertension due to overproduction of renin^37,38^ or blood flow defects related to defective arteriogenesis^5^—from Cx40's specific roles in vasculogenesis and sprouting angiogenesis.

Using the VMO, we found that—similar to the phenotypes reported in Cx40^−/−^ mice^15,16,18^—si-Cx40 microvessel networks (*n* = 18) appeared less complex versus si-Ctrl networks (*n* = 18) when imaged every 2 days following initial cell seeding (Fig. 1C). This was quantified as a subtle but significant reduction in total vessel area in si-Cx40 microvessel networks at days 2 and 6 in the VMO (Fig. 1D), a timepoint at which networks mostly undergo vasculogenesis and sprouting angiogenesis, along with a corresponding increase in mean lacunarity at that timepoint (Fig. 1E). Total vessel length was also significantly decreased at day 2, but not at day 6 (Fig. 1F). Surprisingly, we also noticed a significant and transient increase in branch point number in si-Cx40 networks at day 4 (Fig. 1G), when vessel growth in the VMO is likely supported by both vasculogenesis and angiogenesis. This unexpected finding led us to further explore the specific requirement of Cx40 for sprouting angiogenesis.

### Cx40-deficiency enhances sprouting angiogenesis in vitro

To test involvement of Cx40 in sprouting angiogenesis, we took advantage of the fibrin gel bead assay (“Bead Assay”), an *in vitro* model that specifically recapitulates sprouting angiogenesis.^21–23^ In brief, EC are cultured onto the surface of collagen-coated beads and embedded in a 3D fibrin hydrogel in the presence of primary human fibroblasts. For these studies, fibroblasts were cultured on the hydrogel surface to produce crucial soluble factors that support angiogenesis, while eliminating any potentially confounding factors related to EC-fibroblast cell–cell contact.^22^

**FIG. 2.**
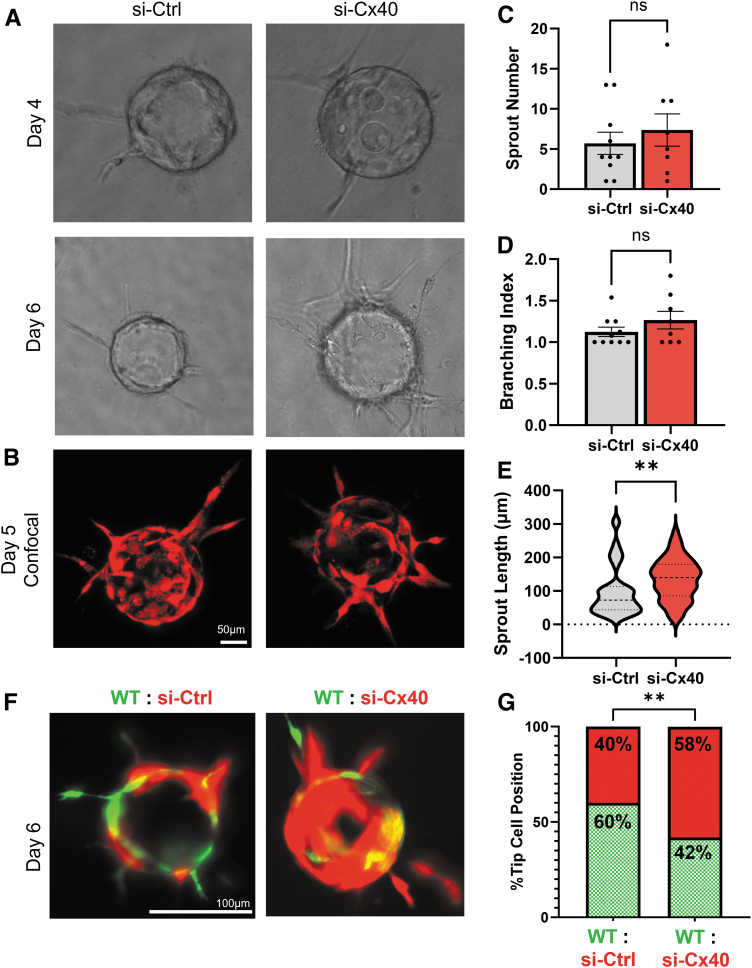
Cx40-deficiency promotes sprouting angiogenesis. **(A)** si-Ctrl and si-Cx40 fluorescent EC were subjected to the Bead Assay model of sprouting angiogenesis, where si-Cx40 EC appeared to undergo more aggressive sprouting. **(B)** A maximal projection of confocal imaging of si-Ctrl and si-Cx40 EC in the Bead Assay at day 5. **(C)** Sprout number per bead and **(D)** sprout branching index were not significantly different between si-Ctrl versus si-Cx40. **(E)** Sprout length was significantly increased in si-Cx40 EC. **(F)** si-Ctrl or si-Cx40 fluorescent EC (red) were co-mixed in equal proportion with wild-type (untransfected) EC expressing a different fluorescent reporter (green). **(G)** si-Ctrl EC occupied the tip cell position in 40% of sprouts, whereas si-Cx40 EC occupied the tip cell position in 58% of sprouts. **p < 0.01.

Over the course of 7 days, EC underwent sprouting angiogenesis—including dynamic acquisition of tip and stalk cell states—to form elongated angiogenic sprouts that radiated from the collagen-coated bead. Interestingly, we observed that si-Cx40 EC appeared to be more robust at undergoing angiogenesis compared to si-Ctrl cells, both in phase/epifluorescence imaging (Fig. 2A) and by maximal projection following confocal microscopy (Fig. 2B). Although neither sprout number per bead (Fig. 2C) nor branching index (Fig. 2D) achieved statistical significance, sprout length was significantly increased with Cx40 knockdown (Fig. 2E).

To confirm that si-Cx40 EC are more pro-angiogenic, we next co-seeded wild-type (i.e., untransfected) mCherry-expression fluorescent EC (green) with equal numbers of either Azurite-expressing fluorescent si-Ctrl or si-Cx40 EC (red). In wild-type (WT): si-Ctrl conditions, both WT and si-Ctrl EC are Cx40-intact and were found to be similarly distributed at the tip cell position of angiogenic sprouts (Fig. 2F, G). By contrast, when si-Cx40 EC were co-mixed with WT EC, they were more abundantly incorporated into angiogenic sprouts (Fig. 2F) and occupied the tip cell position in 61% of quantified sprouts (total *n* = 174 sprouts) (Fig. 2G). This was significantly increased compared to control experiments, where si-Ctrl EC only occupied the tip cell position in 40% of quantified sprouts (total *n* = 135) (Fig. 2G). Thus, si-Cx40 EC outcompete Cx40-expressing WT EC during sprouting angiogenesis.

### Cx40 regulates EC proliferation and migration

**FIG. 3.**
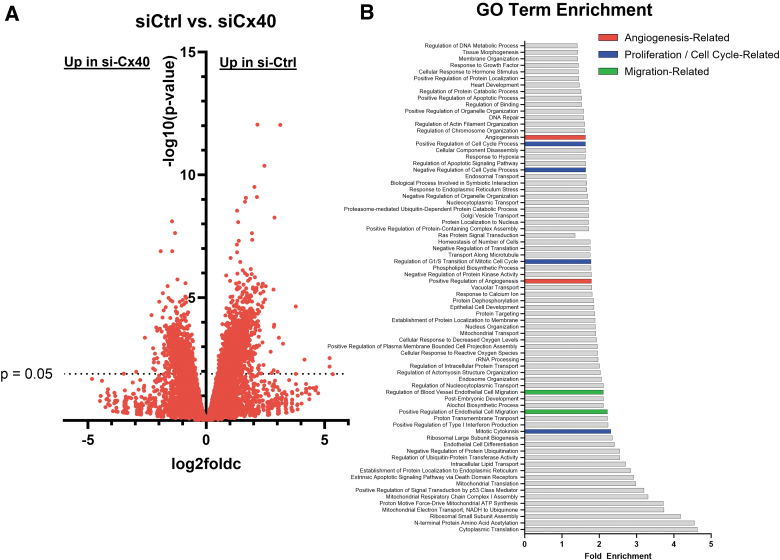
Cx40 regulates angiogenesis, proliferation, and migration. **(A)** si-Ctrl and si-Cx40 EC were compared by bulk RNA sequencing, and 3,065 genes were found to be significantly differentially regulated between these two groups. **(B)** GO term analysis of the DEGs in **(A)** identified 96 GO terms with significant fold enrichment (top 75 displayed), including GO terms related to angiogenesis (red), proliferation (blue), and migration (green). Full DEG list is included with this article as Supplementary Data S1. DEG, differentially-expressed gene; GO, gene ontology.

To better understand the EC functions regulated by Cx40 to control sprouting angiogenesis, we performed transcriptomic analysis of si-Ctrl versus si-Cx40 EC and found that expression of 3,065 genes was significantly different between these two groups (Fig. 3A and Supplementary Data S1), including increased expression of known angiogenic regulators bone morphogenetic protein 4 (BMP4), platelet-derived growth factor (PDGF), and vascular endothelial growth factor B (VEGF-B), transforming growth factor-β (TGF-β), and C-X-C Chemokine Receptor Type 4 (CXCR4) in si-Cx40 versus si-Ctrl. GO analysis on the differentially expressed gene list identified 96 GO terms with a significant fold enrichment, including GO terms related to angiogenesis (red), as well as the cell functions that underlie sprouting angiogenesis: cell proliferation (blue) and migration (green) (Fig. 3B).

**FIG. 4.**
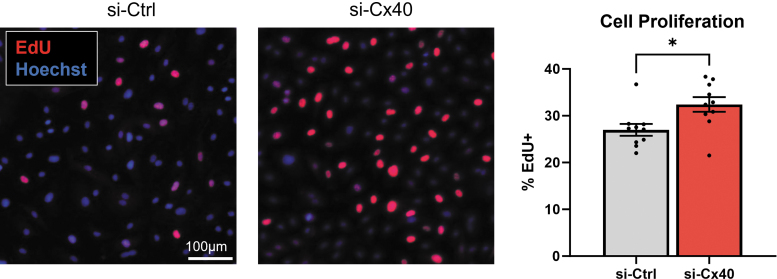
Cx40 knockdown induces EC proliferation. EdU incorporation was compared between si-Ctrl and si-Cx40 EC in monoculture, and percentage of EdU-positive cells was significantly increased for si-Cx40 EC. *p < 0.05. EdU, 5-ethyl-2′-deoxyuridine.

Consistent with the work of Denis et al. who showed increased staining of PCNA (a proliferation marker) with Cx40 knockdown in bEnd3 EC and increased pH3 staining in Cx40 knockout in zebrafish,^6^ we also observed PCNA expression to be increased in si-Cx40 EC by RNA Sequencing (see full list of DEGs, Supplementary Data S1). This—as well as the overall enrichment of GO terms related to cell proliferation—led us to investigate Cx40's possible regulation of EC proliferation. We plated si-Ctrl or si-Cx40 EC in subconfluent monolayers. Then, following a 2.5 h pulse with 20 μM EdU, we fixed and stained cells to assess the extent of EdU incorporation into proliferating cells and found a significant increase in EC proliferation with Cx40 knockdown (Fig. 4).

**FIG. 5.**
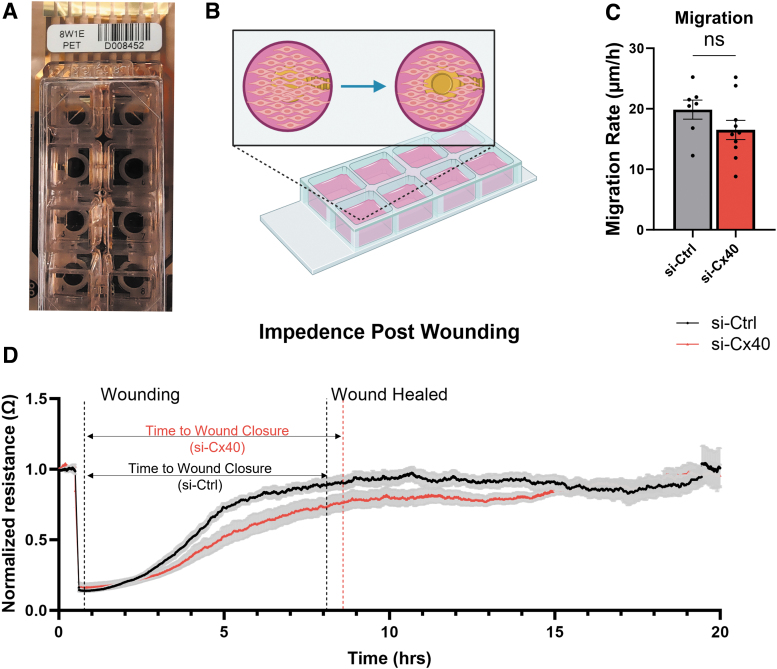
Cx40 does not affect EC migration rate. **(A)** An 8-well ECIS chip containing 0.5 mm^2^ electrode per well was used **(B)** to introduce a focal wound and measure subsequent EC migration to support wound closure. **(C)** There was no significant difference in the rate of migration for si-Ctrl versus si-Cx40 EC, as calculated by **(D)** comparing mean time to wound closure (i.e., time after wounding until resistance plateau, dashed lines). ECIS, electrical cell-substrate impedance sensing.

Next, we used an electrical cell-substrate impedance sensing (ECIS) system, which allows for real-time quantitative measurement of EC migration into a wounded monolayer. Using this approach, we first measured monolayer impedance before wounding. We then generated a focal wound to the monolayer through the introduction of a transient current at the site of the electrode and assessed recovery of monolayer impedance to baseline levels over time. Using this approach, we detected no significant difference in time to wound closure or EC migration rate with Cx40 knockdown (Fig. 5). Interestingly, although time for wound closure was unchanged between si-Ctrl versus si-Cx40 groups, we noticed that overall monolayer resistance was reduced in si-Cx40 EC following wounding even after plateau (Fig. 5D). This suggests that there may be other possible effects of Cx40 knockdown on recovery of cell–cell junctions or cell-substrate adhesion that are of potential interest but beyond the scope of the current study.

### Cx40 maintains Cx37 which suppresses sprouting angiogenesis

**FIG. 6.**
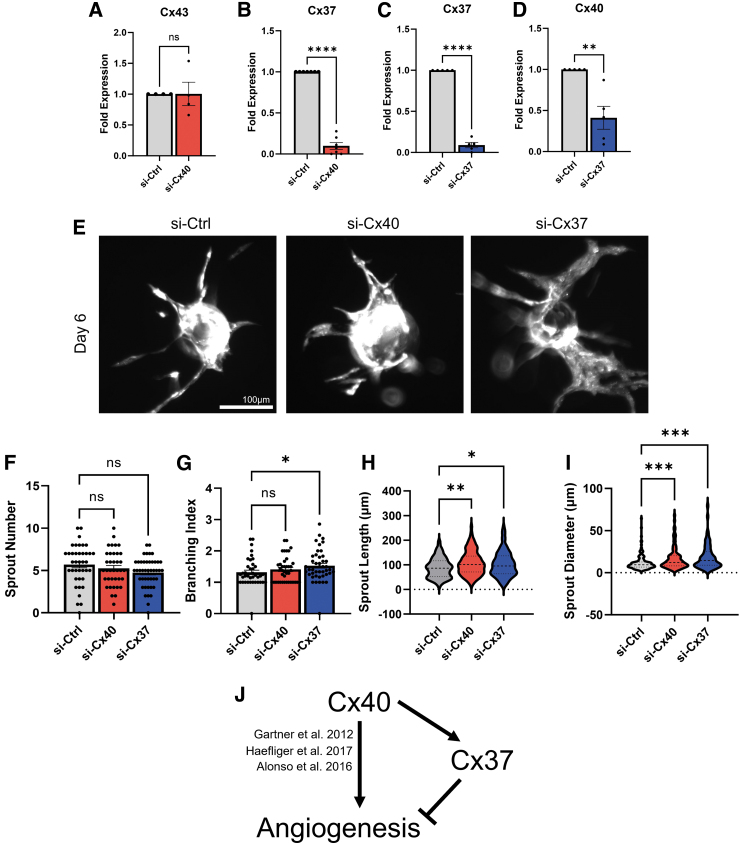
Cx40-knockdown reduces Cx37 expression, which produces a hypersprouting pro-angiogenic phenotype *in vitro*. **(A)** Cx43 transcript expression is unaffected by si-Cx40. **(B)** Cx37 transcript expression is significantly reduced in si-Cx40 EC. **(C)** si-Cx37 significantly reduces Cx37 to similar levels as in si-Cx40, while **(D)** Cx40 transcript levels are only partially knocked down. **(E)** si-Cx40 and si-Cx37 EC produce a phenotype compared to si-Ctrl in the Bead Assay. **(F)** No differences in sprout number per bead were detected, but **(G)** si-Cx37 produces a significant increase in sprout branching index. **(H)** Sprout length is significantly increased in both si-Cx40 and si-Cx37 versus si-Ctrl. **(I)** Sprout diameter is significantly increased in both si-Cx40 and si-Cx37 versus si-Ctrl. **(J)** We therefore propose that in addition to reported effects to promote vascularization, Cx40 may also specifically suppress sprouting angiogenesis through stabilization of Cx37. *p < 0.05, **p < 0.01, ***p < 0.001, ****p < 0.0001.

We have previously shown that Cx37 is potently growth suppressive in EC and in cancer cells through regulation of cell cycle regulator p27,^7,14,39^ and that endothelial expression of this Cx suppresses developmental and postischemic angiogenesis.^7,40,41^ Cx40 is also coexpressed with Cx37 in the larger vessels of many vessel beds,^2,3,7^ where it promotes and stabilizes Cx37 expression.^3,13^ Thus, we hypothesized that Cx40 may regulate sprouting angiogenesis, in part, through effects on Cx37 levels. We found that although Cx43 transcript expression was similar between si-Ctrl and si-Cx40 EC (Fig. 6A), Cx37 transcript levels were almost completely undetectable in si-Cx40 EC (Fig. 6B).

We compared sprouting angiogenesis between si-Cx40 and si-Cx37 EC, in which Cx37 is selectively knocked down (Fig. 6C) while Cx40 expression remains present, although reduced by ∼60% (Fig. 6D). si-Cx37 EC produced an even more exaggerated pro-angiogenic phenotype compared to si-Cx40 EC (Fig. 6E). While sprout number remained unchanged (Fig. 6F), branching index was significantly increased in si-Cx37 EC (Fig. 6G). Sprout length was significantly increased for both si-Cx40 and si-Cx37 compared to si-Ctrl EC (Fig. 6H). We also noticed that si-Cx40 and si-Cx37 sprouts both appeared to be enlarged and dilated compared to si-Ctrl and that this appeared to be especially true for si-Cx37 where we saw angiogenic sprouts composed of sheets of EC (Fig. 6E). To confirm this, we quantified sprout diameter and found sprouts to be significantly dilated for both si-Cx40 and si-Cx37 EC (Fig. 6I).

We interpret our results as indicating that Cx40 specifically suppresses sprouting angiogenesis by limiting EC proliferation, but not migration. We further show that targeted knockdown of Cx40 reduces Cx37 and that Cx37 knockdown alone leads to a disorganized, hypersprouting phenotype *in vitro*. We therefore hypothesize that in addition to any growth-promoting and vessel-stabilizing effects, Cx40 suppresses sprouting angiogenesis and that this may occur at least, in part, through regulation of endothelial Cx37 expression (Fig. 6J).

## Discussion

Prior studies show that Cx40-deficiency leads to reduced microvascular complexity in Cx40^−/−^ mice during development and in cancer,^15,16,18^ but knockout of both Cx40 orthologs in zebrafish embryos significantly enhances intersegmental vessel growth, a process that occurs primarily through sprouting angiogenesis.^6^ In this study, we corroborate the phenotype of Cx40^−/−^ mice using an *in vitro* microvessel-on-a-chip (VMO) model, in which we show that siRNA-based Cx40 knockdown leads to modest reductions in microvessel network complexity.

In intact tissue, as well as in the VMO, vessel density is established through several distinct processes, including vasculogenesis, sprouting angiogenesis, vessel remodeling, and regression. Indeed, studies of Cx40 knockdown in the Matrigel tube-forming assay—a classic model of vasculogenesis—confirm that Cx40 knockdown inhibits this process.^17^ By contrast, however, we find that although Cx40-deficiency leads to an overall reduction in microvessel complexity in the VMO, Cx40-knockdown produces a specific, *hypersprouting* phenotype in an *in vitro* model of sprouting angiogenesis. This was observed when Cx40 was knocked down in all EC, leading to a significant increase in total sprout length (Fig. 2A–E), as well as when Cx40-knockdown EC were allowed to compete with wild-type (Cx40-intact) cells for the tip cell position of individual angiogenic sprouts (Fig. 2F). Taken together, these experiments show that despite overall effects of Cx40-deficiency to reduce vessel complexity, Cx40 knockdown enhances sprouting angiogenesis.

Sprouting angiogenesis depends upon endothelial tip cell migration to drive sprout invasion into avascular tissue, as well as endothelial stalk cell proliferation to support the expanding angiogenic sprout.^42^ Although Haefliger et al. reported that Cx40-knockdown in microvascular EC reduces proliferation,^16^ Denis et al. showed that Cx40 silencing in bEnd.3 increases staining of PCNA (a proliferation marker) with a concomitant reduction of cells in G0/G1.^6^ Denis et al. further confirmed this finding by showing increased pH3 staining (another cell proliferation marker) in the intersegmental vessels of Cx40 knockout zebrafish embryos. Consistent with the findings of this latter study, we also found that Cx40-knockdown in human umbilical vein endothelial cells—an EC type of large vessel origin that is capable of undergoing arteriovenous specification in culture^7^—increases EC proliferation (Fig. 4), which likely supports the hypersprouting phenotype we and others^6^ observe in the absence of Cx40 expression.

Using ECIS for real-time, quantitative measurement of monolayer wounding and recovery, we found that Cx40 knockdown does not alter the speed of EC migration (Fig. 5). This was unexpected given our bulk RNA sequencing analysis in which GO terms for migration were significantly enriched in si-Cx40 versus si-Ctrl (Fig. 3B), but consistent with previous studies that have linked Cx regulation of EC migration to Cx43, not Cx40. For example, an early study showed that forced expression of dominant negative-Cx43 mutants—which broadly reduces cell–cell coupling—inhibits EC migration in a similar monolayer wounding assay.^43^

More recently, Mannell et al. showed that knockdown of endogenous Cx43 alone in human microvascular EC was sufficient to limit EC migration.^44^ Thus, Cx40's pro- and anti-growth effects in the vasculature likely occur primarily through regulation of EC cycle and proliferation, not migration. Interestingly, we did see a lowered overall resistance postwounding in si-Cx40 cells versus si-Ctrl, which may be a result of disrupted cell–cell junctions (or point to a possible role for Cx40 in cell adhesion); further studies are necessary to better understand this observation.

It is currently unclear what might explain the apparently opposing roles for Cx40 in vascularization versus angiogenesis. One possibility is that Cx40 induces distinct (pro- vs. anti-) angiogenic responses based on EC type such that it is pro-angiogenic in endothelium of certain vessel beds but anti-angiogenic in others.

Another possibility is that Cx40's effect on angiogenesis relies on the presence of Cx37, a Cx that is often coexpressed with—and coregulated by—Cx40 in large vessels^2,3,7^ but which has not been reported in microvascular capillary EC. Consistent with this idea, we found that Cx40-knockdown leads to nearly complete loss of Cx37 transcript in our EC. This observation is also consistent with the work of several other groups that report that Cx40 and Cx37 are coregulated and that Cx37 expression is downregulated in Cx40^−/−^ mice.^3,13,45^ Indeed, our data suggest that Cx40 may transcriptionally regulate Cx37, although the mechanism of this is currently unclear. Cx can indirectly upregulate target gene expression through activation of transcription factor intermediaries.^14^ Additional studies are needed to explore if Cx40 might regulate Cx37 gene expression through such a mechanism.

Targeted knockdown of Cx37 phenocopies the effect of Cx40 knockdown in the Bead Assay (Fig. 6). Interestingly, however, the phenotype for Cx37-deficient EC appears more aggressive compared with Cx40-deficient EC, with Cx37 knockdown producing especially enlarged and dilated sprouts composed of EC organized in sheets rather than as narrow, elongated sprout structures (Fig. 6E). This is highly reminiscent of the hyperdense, sheet-like microvascular phenotype observed in the neonatal retinas of Cx37^−/−^ mice,^7,46^ but which does not occur in Cx40^−/−^ animals^16^ or in the VMO (Fig. 1). Indeed, despite the more disorganized appearance of si-Cx37 sprouts, Cx37^−/−^ and Cx40^−/−^ mice remain viable and only mice that lack both Cx37 and Cx40 in combination die perinatally.^10^ This suggests that residual expression of the nonablated Cx in single knockout mice is able to preserve vessel function. Furthermore, given the relatively ordered appearance of si-Cx40 angiogenic sprouts, absence of both Cx in Cx37/Cx40-double knockout animals may disrupt other developmental vascular processes besides sprouting angiogenesis leading to perinatal animal death.

Of further interest is the possibility that single ablation of Cx40 may not only eliminate the formation of Cx40-homomeric and -homotypic gap junction channels but also may disrupt the formation of mixed gap junction channels composed of both Cx40 and other Cx (such as Cx43).^47–49^ The relative contribution of heteromeric and/or heterotypic gap junction channels to *in vivo* vascular physiology is currently unknown. While our work confirms that loss of Cx40 leads to reduced Cx37 expression and an abnormal angiogenic response, additional studies are required to fully understand how loss of Cx40 functionally impacts Cx37 and other Cx in angiogenesis.

In conclusion, we use a novel human cell-based organ-on-a-chip model to confirm *in vivo* mouse studies^16^ showing that Cx40-deficiency leads to an overall reduction in microvessel density. We also report that despite this phenotype, Cx40-deficient EC are more pro-angiogenic versus Cx40-intact EC, a finding that is consistent with findings in EC culture and in zebrafish.^6^ The effect of Cx40 knockdown on sprouting angiogenesis occurs in association with significantly increased rates of EC proliferation, and with no corollary effect on EC migration rate. Finally, we show that Cx40-deficiency leads to reduced Cx37 and that silencing of Cx37 alone produces a more aggressive hypersprouting phenotype similar to that observed in Cx37^−/−^ mice. Taken together, our data demonstrate that endothelial Cx40 suppresses sprouting angiogenesis.
